# Recurrent epibulbar dermoid cyst treated with amniotic membrane implant a case report

**DOI:** 10.1186/s12893-018-0426-z

**Published:** 2018-11-14

**Authors:** Ma. Luisa Villalón, Ma. De Los Ángeles Leal, José R. Chávez, Eduardo M. Santillán, Ismael Lares-Asseff, Verónica Loera, Laura Valencia, Blanca Camacho, Brenda Alvarado, Vilma Cervantes, Leslie Patrón, Horacio Almanza

**Affiliations:** 1 The Californias’ Children Hospital Ophthalmology Service, Av. Alejandro Von Humboldt 11431, Garita de Otay, 22509 Tijuana, BC Mexico; 2 School of Health Sciences, Valle de Las Palmas, Autonomous University of Baja California, Blvd Universitario 1000, Valle de Las Palmas, 22260 Tijuana, BC Mexico; 3 Fray Junípero Serra Hospital, Security and Social Services Institute for State Workers, Tijuana, Avenida De Las Palmas 1 Col Las Palmas, 22106 Tijuana, BC Mexico; 4 Interdisciplinary Research Center for the Comprehensive Regional Development, Durango Module, Durango, Mexico; 5 National Polytechnic Institute, Sigma Street #119, Fracc. 20 de Noviembre II, C.P. 34220. Año 2009-2010 Durango Dgo., Mexico; 6 Department of Cellular Biology and Tissue Engineering, Faculty of Medicine and Psychology, Autonomous University of Baja California, Av. Universidad no 14418, Parque Industrial Internacional Tijuana, CP. 22390 Tijuana, BC Mexico

**Keywords:** Dermoid cyst, Amniotic membrane, Mitomycin c

## Abstract

**Background:**

The dermoid cyst considered a cystic teratoma derived from embryonic germinal epithelium is a slow-growing benign tumour. Dermoid cysts may occur in the orbital and periorbital region in paediatric patients and are often recurrent. The surgical approach depends upon the site of the lesion, superficial or deep. To our knowledge, this is the first described case of a patient with resection of dermoid cyst treated with human amniotic membrane implant and topical application of 0.02% mitomycin C.

**Case presentation:**

We present a case of a 12-year-old male with a tumour in the superotemporal region of the right eye (RE). Symptoms included decreased visual acuity (VA), burning eye, foreign body sensation, and photophobia of the affected eye. A physical examination detected blepharospasm. Ophthalmic examination of his RE, fingers count from a 1–2 m distance, showed no improvement with pinhole. Visual acuity was 20/20 on the left eye (LE). The bio-microscopic examination confirmed the presence of a tumour mass (15 mm × 12 mm) on the surface of the RE, invading the superotemporal sector. The tumour had a lobulated appearance, a shiny and vascularized surface covered by conjunctiva, a pearlescent-pink colour, a medium consistency, was renitent and painless. An ultrasound scan revealed atrophy of the pigmented retinal epithelium with scleral indentation of the RE. A computed tomography (CT) scan revealed a recurrent lesion consistent with an epibulbar dermoid cyst. Surgical excision of the lesion was performed and a human amniotic membrane (HAM) graft and topical 0.02% mitomycin C (MMC) were applied. Histopathological analysis confirmed the diagnosis of recurrent dermoid cyst.

**Conclusion:**

In this case report, we describe a case of recurrent epibulbar dermoid cyst treated with complete resection; topical MMC and HAM implant with good clinical outcome of the lesion and implant adhesion. Resection of a cyst of the ocular surface is not recommended when a large epibulbar dermoid tissue needs to be resected and no HAM graft is available.

## Background

Dermoid cysts are the most common orbital tumours in paediatric patients [1]. Dermoid cysts are considered a form of congenital cystic teratoma of slow growth formed by epidermal inclusions resulting from defective closure of embryonic facial clefts and sometimes containing structures of other germinal layers. They are also considered to be choristomas which are defined as normal embryonic tissue of abnormal location [1–3]. Dermoid cysts are usually benign lesions that can be found in any subcutaneous location; more than 80% are located in the head, mostly in the orbital and periorbital region, especially near the superior temporal frontozygomatic suture [4]. Dermoid cysts can also be found on the ocular surface in the tarsal or bulbar conjunctiva, they are more frequent in the limbus inferotemporal quadrant, which can affect the cornea, and rarely in the orbital and periorbital region [5]. Dermoid cysts are oval, painless whitish, pink or yellowish lesions, surrounded by a stratified squamous epithelium. Sometimes they have a granular layer and produce keratin which is usually thin and no keratinized. The cyst wall contains skin appendices, including hair follicles, sebaceous glands, sweat glands, nerves and smooth muscle, in addition to mesodermal tissue, such as fibrous tissue, fat and blood vessels [6–8]. Dermoid cysts are by far the most common orbital cystic lesions found in children accounting for 3–9% of all orbital tumours [9–11]. The majority of cases are reported between the age of 15 and 35 [11]. Dermoid cysts symptoms vary depending on their location, age at onset and clinical data described by the patients. Symptoms can range from eye discomfort, such as foreign-body sensation, tearing, redness, proptosis or palpebral ptosis, diplopia, restriction of ocular movements, to presence of ocular injury due to the tumour or decreased vision. In advanced cases they can produce a high degree of corneal astigmatism [12–14].

Since dermoid cysts are soft lesions they rarely cause compressive symptoms such as choroidal folds, venous congestion, or optic neuropathy [15] . Diagnosis is initially clinical, supported by ultrasonography, and imaging. Ultimately a histopathological study confirms the type of tissue involved in the injury. Diagnosis is usually not difficult in small lesions, but in larger and deeper orbital lesions it is necessary to include computed tomography (CT) and magnetic resonance imaging (MRI), which can be particularly useful for monitoring the tumour growth [16]. The treatment of small lesions does not require surgery; treatment is required only when the lesion growth produces clinical manifestations such as visual disturbances. Surgery should be performed before palpebral alterations or deviations from the ocular structures are produced. Since dermoid cysts may occur in various sites it is important to establish whether the cyst is superficial or deep in order to perform the appropriate surgery. The surgical option in the superficial lesion is often an incision in the eyebrow, upper eyelid crease or directly over the lesion. For deep lesions, anterior, lateral or combined orbitotomy is indicated. If possible, complete surgical excision without rupture of cyst is the standard of care [17]. A follow-up of the injury is important as the dermoid cyst growth tends to weaken its wall producing leakage of cyst content, which is usually keratin material, in the adjacent soft tissues producing an acute inflammatory response within the orbit or eyelid causing severe localised anaphylactic reaction [3, 4].

The HAM is an avascular membrane, 0.02 to 0.5 mm thick, composed of a single layer of cuboidal cells and its basal membrane resting on a layer of connective tissue close to the chorion. The amniotic membrane (AM) has proven useful in ocular surface lesions as an alternative treatment in various pathologies of the cornea, conjunctiva, sclera and eyelids [18–20]. The AM facilitates healing with minimal inflammation and scarring by combining its mechanical action with biological factors. The AM promotes cell migration, facilitates cell adhesion, cell differentiation [21–23] and other factors that make it useful in the management of injuries of the ocular surface. Mitomycin C is an antibiotic isolated from the fungus *Streptomyces caespitosus*. Mitomycin C has a significant antitumoral action and has been used to inhibit fibroblasts cell proliferation in the postoperative trabeculectomy [24, 25]. Mitomycin C also prevents recurrence of pterygium after resection and has been proven useful in corneal and conjunctival intraepithelial neoplasia (IEN), acting as an alkylating agent capable of inhibiting the synthesis of Deoxyribonucleic Acid (DNA), in such a way that short time exposure is sufficient to suppress cell proliferation. The use of topical MMC in the postoperative care of pterygium can significantly reduce the incidence of recurrence [26–29]. We report the case of a child diagnosed with a tumour on the surface of his RE invading the conjunctiva in the superior and temporal sectors, and the upper third of the cornea. Ophthalmological examination and imaging studies were performed to determine the extent of the tumour and to confirm the diagnosis of recurrent dermoid cyst. To our knowledge, this is the first described case of a patient with resection of dermoid cyst treated with HAM and topical application of 0.02% MMC.

## Case presentation

**Fig. 1:**
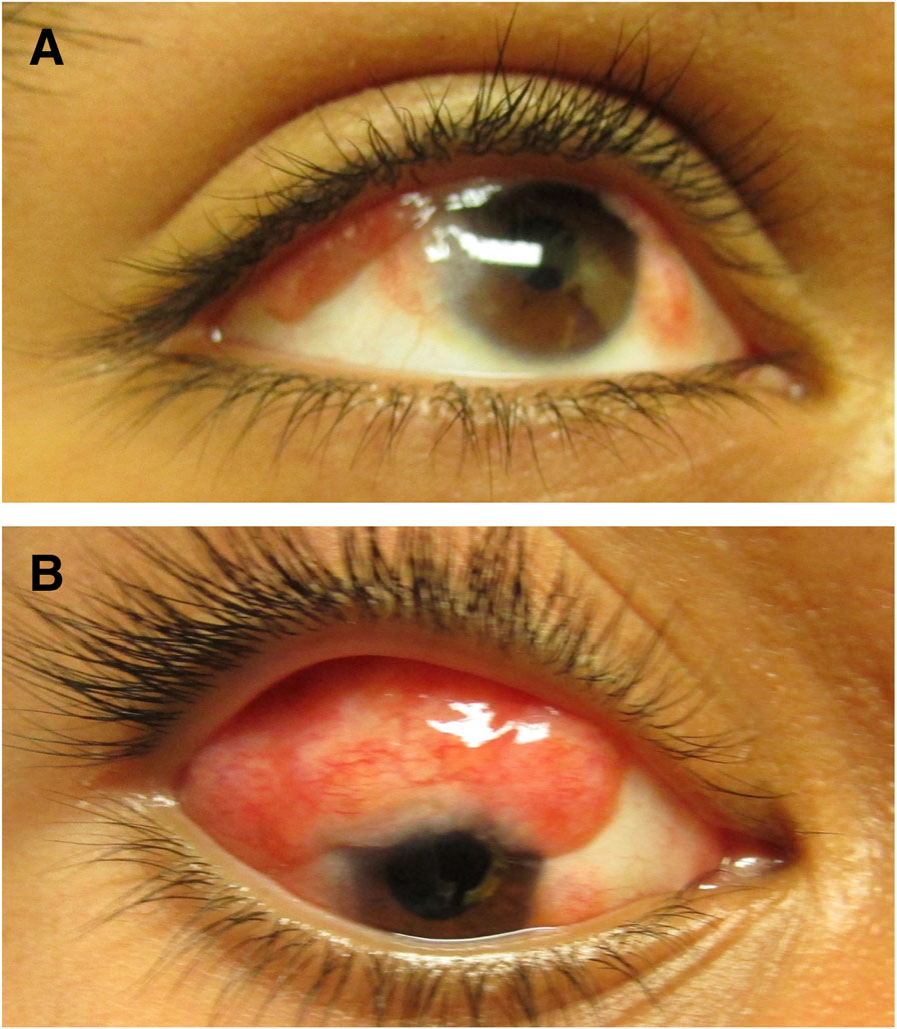
Dermoid cyst

This case was included in a study that adhered to the tenets of the Declaration of Helsinki and was approved by the Ethics Committee of the Faculty of Medicine and Psychology at the Baja California Autonomous University which authorized the use of HAM for the treatment of injuries of the ocular surface. We present a case of a 12-year-old male patient with a tumour in the superotemporal region of the RE, with decreased VA, burning eye, foreign body sensation and photophobia of the affected eye, reporting tumour growth during the last year. Ophthalmological examination of his RE, un-corrected VA and finger count from 1 m distance showed no improvement with pinhole. Visual acuity was 20/20 on the LE (Snellen chart). After the administration of 1% cyclopentolate cycloplegic refraction was performed using a phoropter and retinoscopy. Results were VA + 3.25–3.00 × 175, 20/80 on the RE and VA + 1.75, 20/20 (Snellen chart) on the LE. Palpebral elevation, anterior segment and biocular motility were normal. The bio-microscopic ophthalmological examination confirmed the presence of a tumour mass (15 mm × 12 mm) on the surface of his RE, invading the superior and temporal sector of the eye. The tumour had a lobulated appearance, a shiny vascularized surface covered by conjunctiva, a pearlescent-pink colour, a medium consistency, was renitent and painless. The tumour adhered to deep layers in more than an upper third (Fig. 1A), resulting in asymmetry of the upper eyelid of the RE, which was elevated and had a closed appearance. The function of the upper right eyelid elevator muscle was normal; there were no alterations in the LE.

**Fig. 2:**
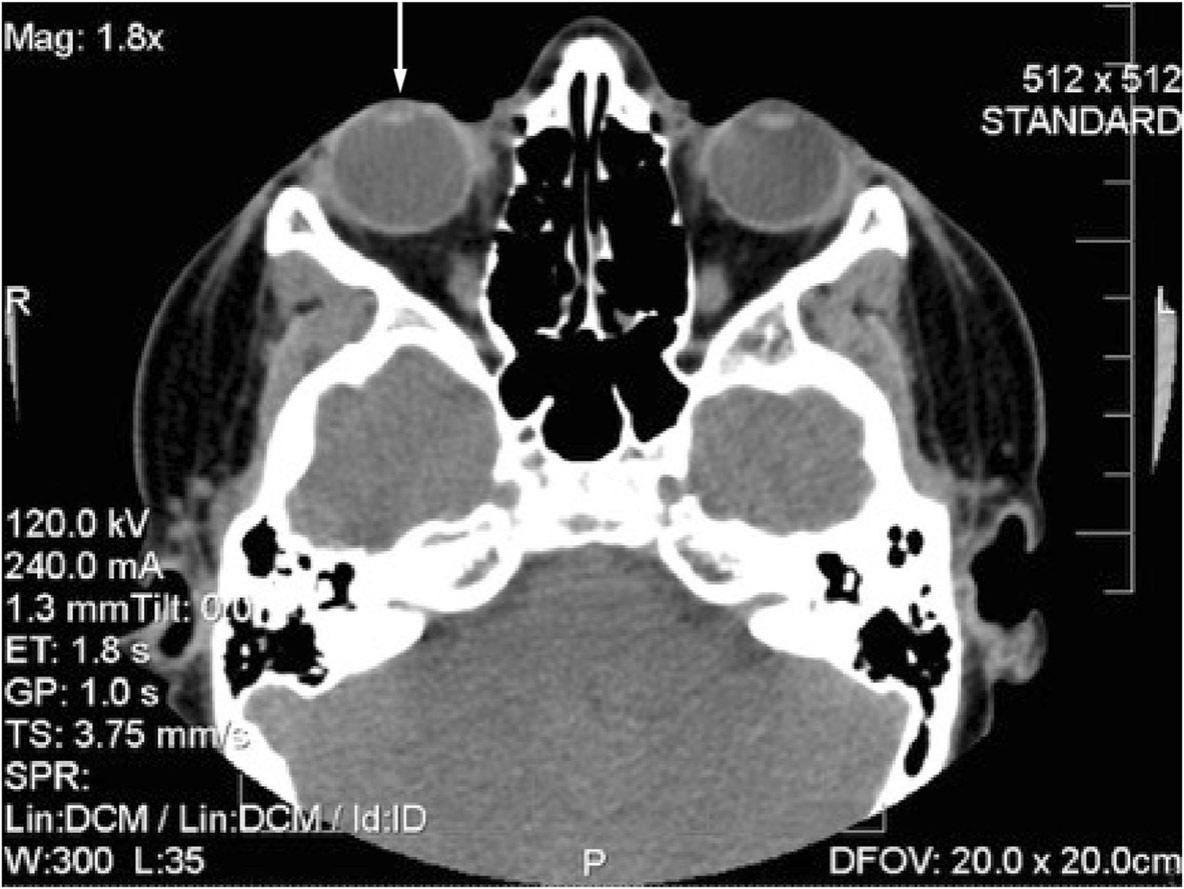
Computed tomography

Isochoric pupils with normal pupillary reflexes were revealed. Wide anterior chamber with normal iris, transparent crystalline, vitreous and homogeneous in eyes, applied retina, normal macula, apparently normal retinal vessels, retinal pigment epithelium (RPE) area of atrophy and upper nasal sector revealing choroidal vessels were evident. No pathological scleral indentation was found. The cycloplegic refraction showed high hypermetropic astigmatism (Fig. 1B). A B-scan ultrasonography revealed a tumour on the eye surface that did not invade the anterior chamber or deep layers of the eyeball. The vitreous and retina showed no damage. A CT scan revealed a recurrent lesion consistent with an epibulbar dermoid cyst without extension to orbit and without orbital rim bone erosion (Fig. 2). It should be noted that the lesion started when the patient was three years old (2006) and was characterized by a tumour mass. No macroscopic characteristics or clinical symptoms of the tumour were provided. An exeresis biopsy was performed to remove the tumour and the histopathological analysis showed the presence of lacrimal acini, adipose tissue and blood cells consistent with a conjunctival dermolipoma. During five years there was no clinical follow-up.

The preparation of the AM was performed using slight modifications of the method described by Kim and Tseng. The human placenta was obtained shortly after elective caesarean delivery in women who were between 18 and 35 years of age. All donors signed an informed consent prior to caesarean delivery. Women had been controlled for at least 6 months, had a healthy foetus without placental pathology and were seronegative for human immunodeficiency virus type 1 and 2, hepatitis B and C, *Treponema pallidum*, *Brucella abortus* and *Trypanosoma cruzi*. Next, in a laminar flow hood, the placenta was cleaned with a sterile phosphate and saline solution containing antibiotic/antimycotic (50 μg/ml of penicillin, 50 μg/ml of streptomycin and μg/ml of amphotericin B) (Sigma-Aldrich Corp, Saint Louis, USA) to remove blood clots. The amnion was separated from the chorion using a blunt dissection and the membrane was cut to 4 × 4 cm. Membrane squares were spread on a nitrocellulose filter paper, placed in a sterile vial containing Dulbecco’s modified Eagle’s medium (Biowest SAS, Nuaillé, France) plus glycerol and human albumin (Sigma-Aldrich Corp, Saint Louis, USA) in a 1:1 ratio at 10% and frozen at − 80 °C. The AM was thawed in a water bath at + 37 °C with saline solution, and samples were taken for microbiological and histological controls [30].

**Fig. 3:**
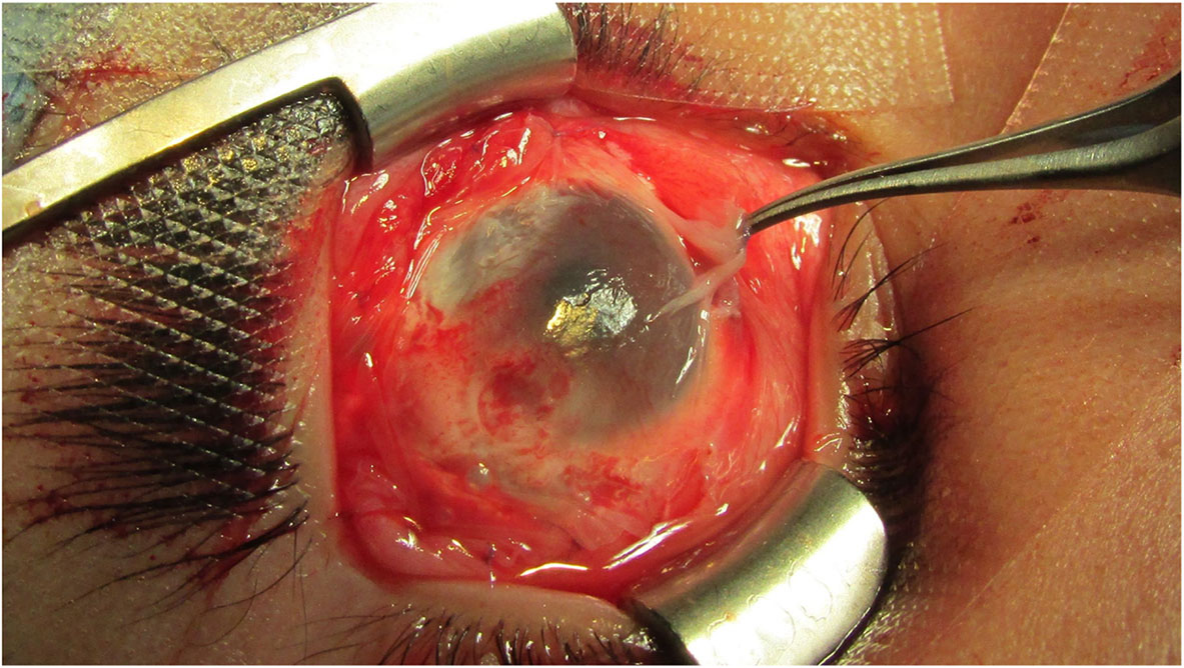
Surgical intervention

Removal of the tumour was performed under general anaesthesia prior preoperative patient preparation and preoperative tests. Xylocaine with epinephrine was injected for vasoconstriction and dissection of adherent tissues. The excision started at the level of the sclero-corneal limbus using Stevens’ scissors and a scalpel blade # 15 and continued toward the bottom of the superior nasal and the superior temporal socket respecting mainly the superior and lateral rectus eye muscles which were free of tumour. The dissection was carefully performed given the adhesions to Tenon and sclera sectors and lamellar keratoplasty of the cornea was performed up to the superficial stroma. 0.02% MMC was applied with a merocel sponge for 3 min and washed profusely thereafter with 10 cc of physiological solution. The stromal side of the AM implant was placed on the conjunctiva and over the entire cornea and sutured with interrupted 7/0 Vicryl stitches in the denuded area up to near the bottom of the superior socked, covering the cornea completely. Eye drops and antibiotic ointment were applied, the eye was patched and the patient was discharged (Fig. 3). The biopsy was sent for a histopathological study, data were obtained from medical records. The dermoid cyst socket, the initial and final vision, the improvement of the corrected VA in both eyes, the presence or absence of symptoms such as irritation, surgical or postoperative complications and good cosmetic result, defined as the absence of any mass or unsightly scars in the primary position of the lesion by external examination, were evaluated at different periods of time.

**Fig. 4:**
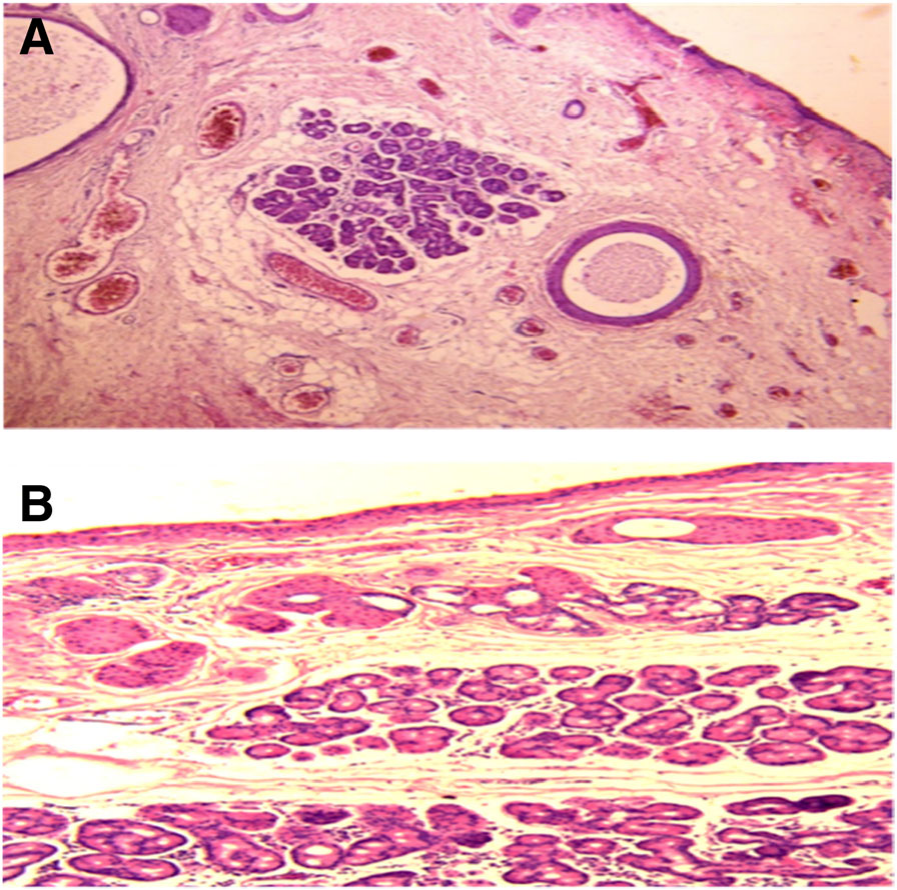
Conjunctival dermoid tumour

**Fig. 5:**
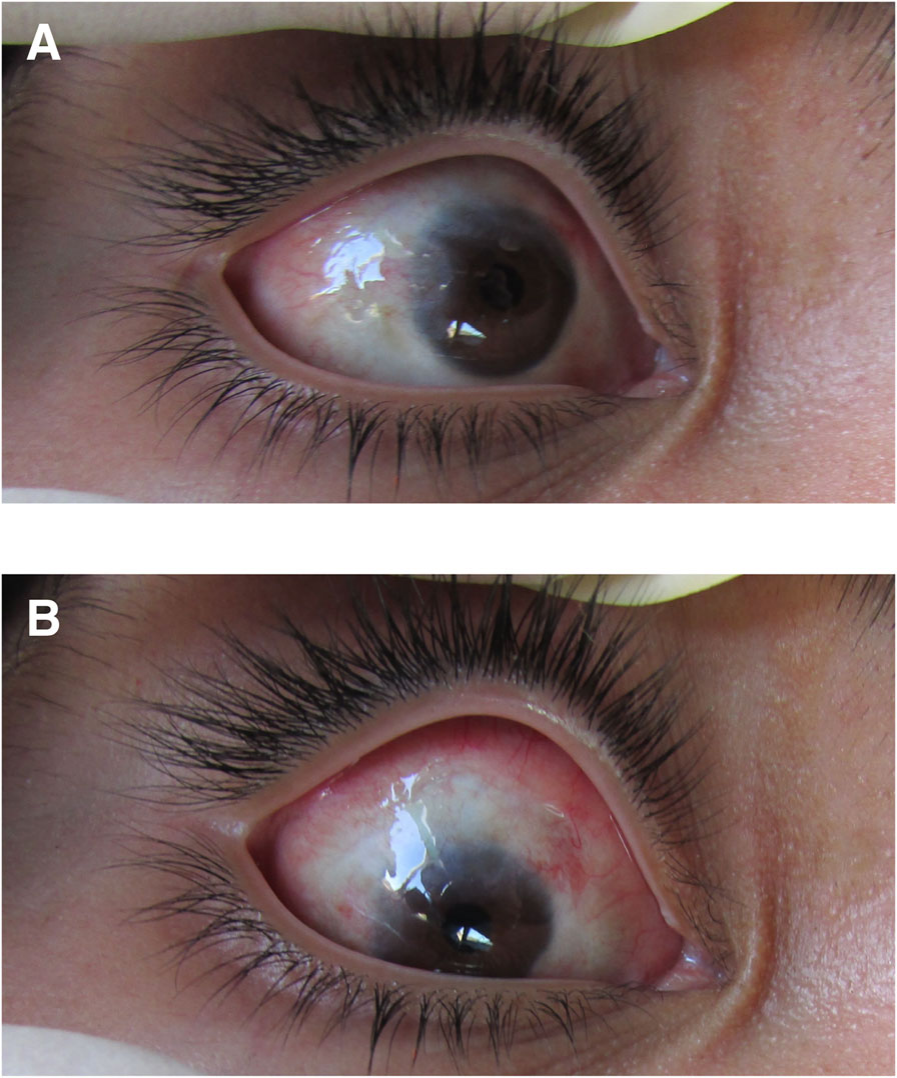
Postoperative appearance

The preoperative study results were normal; the ultrasonography showed a clear image with defined edges, amblyopia of the right eye with atrophy of the superior nasal RPE and diagnosis of conjunctival tumour on the right eye. Computed tomography suggested a recurrent lesion consistent with dermoid cyst. The cystic mass located in the nasal and temporal region of the eyeball upper part, with projection to the bottom of the socket and invasion of the cornea, conjunctiva and parotid duct, required the removal of the lesion through an incision along the lower orbital margin and superficial layers of the cornea with the application of 0.02% MMC during 3 min over the sclera with a merocel sponge and profuse washing with physiological solution. Over the follow-up period to evaluate symptoms and possible infections the patient reported moderate pain during the first 24 to 48 h. During the first week the patient’s symptoms were foreign body sensation, mucous secretion, tearing and blepharospasm. During the second week the cornea appeared opaque due to the coating of the HAM and the patient could only see lumps with his RE. Tobramycin/dexamethasone was applied and cleaning solution and lubricant drops were recommended for a period of one month. The correction of the VA was variable, the RE VA without correction was 20/70 at 9 weeks and 20/50–1 at 5 months. His RE VA without correction was 20/100 at 8 months improving with pinhole to 20/80. At 5 months the cornea was completely clear and the incorporation of the implant into the cornea and sclera was very good. No signs of implant rejection were detected and the right upper eyelid showed normal symmetry. Symptoms decreased and there were no postoperative complications. The visual outcome was moderately good; the cosmetic result was very good, as defined by the absence of significant or unsightly scars in the primary site of the lesion during external examination. There were no adhesions or symblepharon. Several tissue fragments in formalin, designated as tumour of the right eye conjunctiva dependent, were sent for histopathological study. The histopathological report described a piece of tissue with irregularly lobed surface, pale pink colour and soft consistency measuring 2.6 × 0.6 × 0.5 cm. A mixed tumour was identified histologically; this lesion was located within the choristomas (benign neoplasm of heterotopic location). This biphasic neoformation showed mesenchymal tissue, which was represented by loose connective tissue with abundant congestive blood vessels with thin walls and few fat cells. Cystic formations coated with keratinized stratified squamous epithelium containing thin slices of keratins. Groups of sebaceous glands immersed in dense bundles of keratin corresponded to the epithelial component (Fig. 4a). When the lesion was analysed on higher magnification it was cytologically identified that the elements that formed it were well differentiated and had no atypia suggestive of malignancy (Fig. 4b). No visible remains or tumour recurrence as well as scarring in the first year of follow-up were present. The revitalization of the area where the AM was implanted and treated with MMC healed during the first month without any postoperative complication. The results of the clinical course were good injury site healing, subconjunctival haemorrhage and conjunctival inflammation only during the first two weeks of the postoperative and complete absence of infection, necrosis, edema, diplopia, recurrence or symblepharon up to 14th months after the postoperative follow-up confirms the patient good clinical outcome. The patient was satisfied with the cosmetic appearance and with the clinical outcome, giving a high satisfaction score (Fig. 5a and b).

## Discussion and conclusions

Dermoid cysts are by far the most common orbital cystic lesions found in children accounting for 3% to 9% of all orbital tumours [7, 10, 11]. Congenital, formed by epidermal inclusions resulting from the defective closure of embryonic facial clefts they sometimes contain structures of other germ lines. Dermoid cysts produce painless, oval, whitish, pink or yellow colour lesions surrounded by a stratified squamous epithelium. Sometimes they have a granular layer and produce keratin usually thin and no keratinized. The cyst wall contains skin appendages including hair follicles, sebaceous glands, sweat glands, nerves and smooth muscle, along with tissue of mesodermal origin, such as fibrous tissue and blood vessels [6–8]. The treatment of orbital dermoid cysts depends on the location, size and associated anomalies of the cyst. Imaging techniques such as ultrasound, CT and MRI of the dermoid cyst are valuable in the diagnosis and characterization of benign lesions and also to show the actual extent of the injury in the ocular surface [31–33]. Surgical excision can be considered in order to improve the patient’s vision, prevent amblyopia, and eliminate persistent irritation and cosmetic problems. Treatment involves complete removal. It is important to keep the cyst intact during surgery because leaking of the cyst material or persistence of residues, produces an acute inflammatory response [3, 4].

In the last decade, AM transplantation has been applied in a variety of ocular surface problems. The importance of the AM is its ability to reduce inflammation, improve epithelization and wound healing, as well as its anti-angiogenic properties [23]. The AM has been used for the reconstruction of the conjunctiva and resulted in a good cosmetic outcome [18, 19]. The AM has also been widely used in the reconstruction of corneal surface in several injuries such as neurotrophic ulcers [34, 35], persistent epithelial defects, microbial keratitis [36, 37], band keratopathy, bullous keratopathy [38, 39] and chemical injuries [38, 40]. Due to its properties, an AM implant was used in this 12-year-old patient with a recurrent dermoid cyst. An excellent clinical outcome was obtained throughout the 14 months period of medical follow-up.

In conclusion, we describe what may be the first report of a case of a recurrent dermoid cyst in the nasal and temporal region of the bulbar conjunctiva with invasion of cornea and subsequent alteration of the palpebral position and vision due to high hypermetropic astigmatism, treated successfully with excision of the lesion, application of topic 0.02% MMC, profuse washing and placement of HAM as a substitute conjunctiva and portions of Tenon’s capsule as well as superficial layers of the cornea. Medical monitoring at different times of surgical response and implant application showed good progress of the injured site. The histopathological exam revealed a cystic formation covered with keratinized stratified squamous epithelium containing thin layers of keratin and groups of sebaceous glands immersed in dense bundles of keratin that correspond to the epithelial component (Fig. 4a). There was no tumour recurrence and scarring during the first 14 months of follow-up and the patient was satisfied with the cosmetic appearance and the clinical course. His VA improved substantially without corrective lenses (Snellen) counting fingers from a 1 m distance 10/80. The patient follow-up will continue to evaluate recurrence, changes in scleral thickness and VA.

**Fig. 6:**
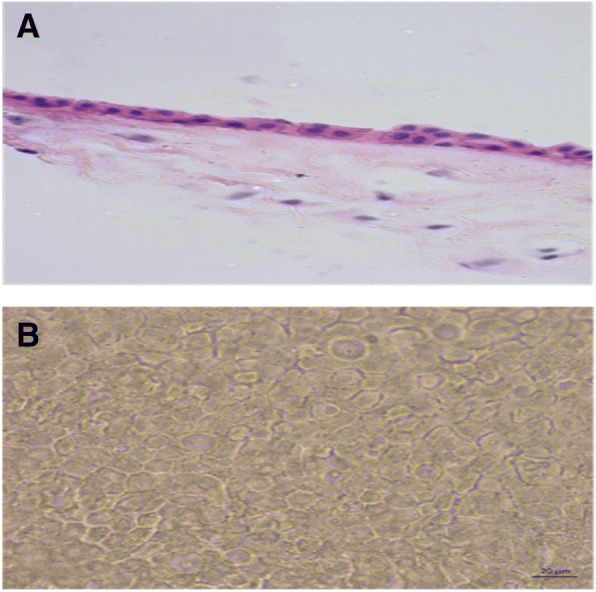
Histological structure of the AM cryopreserved with glycerol and albumin in Dulbecco’s modified Eagle’s medium at − 80° C

In the study reported by Matsuo, the clinical characteristics of superficial ocular dermoid cysts in paediatric patients [40] were evaluated in order to offer the best option for a clinical decision-making; either resection or observation of the cyst growth to determine the appropriate time for the excision. In this study we showed the efficacy and safety of the AM implant with MMC application to repair conjunctival defects after removal of the dermoid cyst. We also concluded that the extent and depth of the tumour should be determined by ultrasonography and CT before surgical resection, since these tumours may have a clinically superficial appearance, but imaging and ultrasound can show the actual extent and depth of the lesion creating knowledge base for decision-making. Consequently, in this case and given the extent of the injury reported by the tests, the surgical management employed with application of AM graft and MMC proved to be a good treatment alternative for dermoid cyst extensive injuries. This coupled with the changes made in the AM cryopreservation that maintain the normal histological structure and thereby (Fig. 6a and b) its anti-inflammatory properties, promoting cellular migration facilitates cellular adhesion, cellular differentiation [21, 23] and scarring. Finally, this study recommends not performing surgical resection on a cyst of the ocular surface when a large epibulbar dermoid tissue needs to be resected and no AM graft is available.

## Abbreviations

AM: Amniotic Membrane
CT: Computed Tomography
DNA: Deoxyribonucleic Acid
HAM: Human Amniotic Membrane
IEN: Conjunctival Intraepithelial Neoplasia
MMC: Mitomycin C
MRI: Magnetic Resonance Imaging
RPE: Retinal Pigment Epithelium
VA: Visual Acuity
